# Retained capacity for perceptual learning of degraded speech in primary progressive aphasia and Alzheimer’s disease

**DOI:** 10.1186/s13195-018-0399-2

**Published:** 2018-07-25

**Authors:** Chris J. D. Hardy, Charles R. Marshall, Rebecca L. Bond, Lucy L. Russell, Katrina Dick, Cono Ariti, David L. Thomas, Sonya J. Ross, Jennifer L. Agustus, Sebastian J. Crutch, Jonathan D. Rohrer, Doris-Eva Bamiou, Jason D. Warren

**Affiliations:** 1 Dementia Research Centre, Department of Neurodegenerative Disease, UCL Institute of Neurology, Queen Square, London, WC1N 3BG UK; 2 London School of Hygiene and Tropical Medicine, London, UK; 3 Neuroradiological Academic Unit, Department of Brain Repair and Rehabilitation, UCL Institute of Neurology, London, UK; 4 Leonard Wolfson Experimental Neurology Centre, UCL Institute of Neurology, London, UK; 5 UCL Ear Institute and UCLH Biomedical Research Centre, National Institute for Health Research, London, UK

**Keywords:** Sinewave speech, Degraded speech, Perceptual learning, Dementia, Progressive aphasia, Semantic dementia, Alzheimer’s disease, VBM, Progressive non-fluent aphasia, Logopenic aphasia

## Abstract

**Background:**

Processing of degraded speech is a promising model for understanding communication under challenging listening conditions, core auditory deficits and residual capacity for perceptual learning and cerebral plasticity in major dementias.

**Methods:**

We compared the processing of sine-wave-degraded speech in 26 patients with primary progressive aphasia (non-fluent, semantic, and logopenic variants), 10 patients with typical Alzheimer’s disease and 17 healthy control subjects. Participants were required to identify sine-wave words that were more predictable (three-digit numbers) or less predictable (place names). The change in identification performance within each session indexed perceptual learning. Neuroanatomical associations of degraded speech processing were assessed using voxel-based morphometry.

**Results:**

Patients with non-fluent and logopenic progressive aphasia and typical Alzheimer’s disease showed impaired identification of sine-wave numbers, whereas all syndromic groups showed impaired identification of sine-wave place names. A significant overall identification advantage for numbers over place names was shown by patients with typical Alzheimer’s disease, patients with semantic progressive aphasia and healthy control participants. All syndromic groups showed spontaneous perceptual learning effects for sine-wave numbers. For the combined patient cohort, grey matter correlates were identified across a distributed left hemisphere network extending beyond classical speech-processing cortices.

**Conclusions:**

These findings demonstrate resilience of auditory perceptual learning capacity across dementia syndromes, despite variably impaired perceptual decoding of degraded speech and reduced predictive integration of semantic knowledge. This work has implications for the neurobiology of dynamic sensory processing and plasticity in neurodegenerative diseases and for development of novel biomarkers and therapeutic interventions.

**Electronic supplementary material:**

The online version of this article (10.1186/s13195-018-0399-2) contains supplementary material, which is available to authorized users.

## Background

Deficits of speech perception have been documented in the three major ‘language-led’ dementias (non-fluent variant primary progressive aphasia [nfvPPA], semantic variant PPA [svPPA] and logopenic variant primary progressive aphasia [lvPPA]) [1–5] as well as in typical Alzheimer’s disease (tAD) [6–9]. However, the factors that affect speech perception have been less well studied than language output in these diseases. Normal speech perception entails high-fidelity encoding of incoming acoustic data, parsing of speech from extraneous noises and integration with prior expectations [10]. Healthy listeners rapidly and automatically adapt to speech degradation under challenging listening conditions, based on prior auditory experience [11]. This reflects perceptual learning: improved accuracy of perceptual processing following sustained exposure to the auditory stimulus, modulated by ‘top-down’ predictive mechanisms [12, 13].

Perception of degraded speech is impaired in patients with nfvPPA, svPPA and tAD [14, 15], whereas implicit auditory sequence learning and disambiguation of degraded speech are retained in nfvPPA [10, 16], albeit with reduced flexibility in using contextual information. Processing degraded sensory stimuli taxes the functional integrity of cortical circuits [17, 18], and disorders such as PPA and tAD constitute test cases for exposing such effects because they strike auditory processing networks early and relatively selectively. In contrast, perceptual learning reflects functional brain reorganisation or plasticity; it might therefore help compensate for effects of neurodegeneration [19]. Adaptation to degraded speech engages areas (such as sensorimotor cortex) beyond classical language and auditory networks [20, 21] and may be enhanced by cholinergic stimulation [15]. Taken together, these considerations suggest that understanding and perceptual learning of degraded speech might constitute a powerful and sensitive probe of neural network integrity and residual plasticity in neurodegenerative syndromes.

In the present study, we assessed identification of degraded speech and associated auditory perceptual learning in patients with PPA and tAD relative to healthy older individuals. We used sine-wave speech as a model paradigm. Sine-wave speech is a radical perceptual alteration that reduces speech signals to a series of ‘whistles’ that correspond to formant contours, retaining the long-range temporal scaffold of speech but stripped of all spectral detail (examples are available in the additional files). Sine-wave transformation renders speech initially unintelligible, yet induces spontaneous perceptual learning in healthy listeners primed to its linguistic origin [11, 22]. This effect relies on ‘top-down’ perceptual integration of apparently dissimilar acoustic events into a coherent speech-like signal. It is therefore likely a priori to depend on cognitive operations that are instantiated across the language network. To explore the effect of semantic predictability on the ‘top-down’ disambiguation of degraded speech [10], we applied sine-wave manipulations to a verbal category with uniformly high predictability (numbers) and a verbal category in which predictability could be varied on the basis of familiarity (geographical place names). These semantic categories are relatively preserved in svPPA [2, 23], allowing predictive processing mechanisms to be distinguished from semantic disintegration. Neuroanatomical associations of sine-wave speech processing in the patient cohort were assessed using voxel-based morphometry (VBM).

On the basis of available evidence [3–5, 10, 15, 24–27], we hypothesised that PPA syndromes and tAD would be associated with differential impairment of sine-wave speech identification and perceptual learning relative to controls. We predicted that nfvPPA and lvPPA would be associated with more severe perceptual decoding deficits than other groups, but that all groups would show retained adaptation to degraded speech and increased reliance on prior predictability, particularly in svPPA. Drawing on neuroanatomical evidence in the healthy brain and PPA [10, 13, 20, 28–30], we further hypothesised that sine-wave speech perception deficits would correlate with grey matter loss in posterior superior temporal and inferior parietal cortices, whereas modulation by prior predictability and perceptual learning effects would correlate with anterior sensorimotor, prefrontal and anterior temporal grey matter.

## Methods

### Participants

Nine patients with nfvPPA, ten patients with svPPA, seven patients with lvPPA and ten patients with tAD were recruited via a specialist cognitive clinic. Seventeen healthy older individuals with no history of neurological or psychiatric illness also participated in the study. All patients fulfilled current consensus diagnostic criteria either for the relevant PPA syndrome [1] or for Alzheimer’s disease [31]. Syndromic diagnoses were corroborated by a general neuropsychological assessment (Table 1) and brain magnetic resonance imaging (MRI) findings. No patients had radiological evidence of significant co-morbid cerebrovascular disease. Cerebrospinal fluid profiles of tau and beta-amyloid were available for five of the seven patients with lvPPA and were consistent with Alzheimer’s pathology in each case, based on local reference ranges (total tau/beta-amyloid_1–42_ ratio > 1). No participant had a history of clinically relevant hearing loss; each participant’s peripheral hearing function was assessed using a previously described pure-tone audiometry protocol [14].

**Table 1:** Demographic, clinical and general neuropsychological data for the participant groups

	Controls	nfvPPA	svPPA	lvPPA	tAD
Demographic and clinical					
No. of participants, M/F	8/9	3/6	7/3	6/1	4/6
Age, years	67.7 (5.2)	69.6 (9.2)	64.9 (7.6)	66.3 (6.1)	70.5 (8.9)
Handedness (R/L/A)	16/0/1	8/1/0	10/0/0	7/0/0	9/1/0
Education, years	16.2 (2.6)	14.9 (3.3)	14.8 (3.3)	15.1 (2.3)	14.0 (1.8)
MMSE (total possible score of 30)	29.7 (0.5)	**24.4 (5.1)**	**25.2 (5.3)**	**18.4 (8.0)**	**19.1 (5.1)**
Symptom duration, years	NA	3.6 (1.3)	5.3 (2.0)	3.3 (1.3)	6.1 (3.1)
PTA best ear (N/mild/moderate)	4/11/0^a^	1/6/1^b^	4/6/0	3/2/1^b^	2/5/0^c^
General intellect (IQ)					
WASI Verbal IQ	127.6 (5.9)	**76.4 (17.7)**	**67.5 (22.4)**	**60.6 (8.3)**	**91.8 (19.3)**
WASI Performance IQ	121.7 (13.7)	**100.3 (21.8)**	110.1 (21.8)	**79.4 (13.1)**	**84.7 (20.3)** ^**b**^
Episodic memory					
RMT Words (total possible score of 50)	48.4 (1.9)	**40.3 (7.3)** ^**a**^	**33.4 (5.7)** ^**d**^	**31.0 (7.3)** ^**a**^	**15.7 (3.5)** ^**b,e**^
RMT Faces (total possible score of 50)	44.5 (4.4)	39.1 (4.0)^a^	**35.0 (6.2)** ^**c**^	**31.7 (5.2)**	**18.2 (3.2)** ^**b,e**^
Working memory					
Digit span forward (maximum)	7.2 (1.0)	**4.6 (1.4)**	7.2 (1.2)	**4.0 (1.3)** ^**b**^	**6.2 (0.9)**
Spatial span forward (maximum)	5.5 (0.8)^a^	**4.8 (1.2)**	5.5 (0.9)	**3.3 (0.8)**	NA
Executive skills					
Digit span reverse (maximum)	5.1 (1.1)	**3.0 (0.9)** ^**b**^	5.4 (2.1)	**2.6 (0.9)** ^**a**^	**3.8 (0.8)** ^**b**^
Spatial span reverse (maximum)	5.4 (0.9)^a^	**3.8 (1.5)**	5.2 (1.2)	**3.0 (1.0)**	NA
Letter fluency (total)	18.4 (5.1)	**6.2 (6.0)**	**10.2 (4.5)** ^**b**^	**4.5 (6.5)** ^**c**^	**9.9 (6.0)**
Category fluency (total)	25.6 (5.4)	**9.7 (4.9)**	**17.6 (37.2)** ^**b**^	**5.0 (7.5)**	**6.3 (4.9)**
Trails Making Test A (seconds)	31.8 (8.0)	**71.3 (36.9)**	**45.1 (37.2)**	**79.2 (37.6)** ^**a**^	**92.8 (40.6)** ^**a**^
Posterior cortical skills					
GDA Calculation (total possible score of 24)	13.6 (4.1)	**6.0 (6.4)** ^**b**^	15.0 (7.3)^a^	**3.0 (2.2)** ^**c**^	**3.4 (4.4)** ^**c**^
VOSP Object Decision (total possible score of 20)	18.9 (1.0)	**17.4 (1.9)**	**16.2 (3.1)**	**15.3 (2.6)**	**15.5 (2.3)**
Neurolinguistic skills					
Auditory input processing					
PALPA-3 (total possible score of 36)	35.1 (1.1)^a^	34.6 (2.3)	35.3 (1.0)	**31.1 (5.2)**	NA
Word retrieval					
GNT (total possible score of 30)	27.1 (2.5)	**13.8 (4.8)** ^**a**^	**1.2 (2.2)** ^**d**^	**9.3 (10.3)**	**12.7 (9.2)** ^**b**^
BNT (total possible score of 30)	29.4 (0.6)^a^	**22.0 (5.0)**	**6.4 (5.2)** ^**a**^	**9.9 (8.5)**	NA
Comprehension					
BPVS (total possible score of 51)	48.3 (5.6)	**33.3 (14.9)**	**9.5 (14.8)**	**29.3 (7.3)**	**40.1 (5.5)** ^**b**^
Synonyms concrete (total possible score of 25)	24.5 (0.6)^a^	**19.0 (4.2)** ^**b**^	**16.6 (3.3)** ^**c**^	**17.7 (2.8)**	NA
Synonyms abstract (total possible score of 25)	24.5 (0.8)^a^	**19.3 (4.5)** ^**b**^	**15.6 (3.6)** ^**c**^	**17.8 (4.0)** ^**b**^	NA
PALPA-55 (total possible score of 24)	23.9 (0.4)^a^	**19.1 (4.5)**	22.3 (2.1)^c^	**15.7 (4.9)**	NA
Speech repetition					
Polysyllabic words (total possible score of 45)	44.8 (0.9)^a^	**35.1 (3.6)** ^**b**^	48.9 (0.6)	**34.5 (2.6)**	NA
Short sentences (total possible score of 10)	9.7 (0.6)^a^	**4.0 (2.9)** ^**a**^	**7.8 (1.7)** ^**a**^	**4.6 (2.2)**	NA
Spelling					
BST (total possible score of 30)	26.6 (1.6)^a^	**14.2 (8.0)**	**13.0 (7.5)** ^**b**^	**13.0 (7.3)** ^**b**^	NA

*Abbreviations: A* Ambidextrous, *BNT* Boston Naming Test [52], *BPVS* British Picture Vocabulary Scale [53], *BST* Baxter Spelling Test (Baxter & Warrington, 1994), *Controls* healthy control group, *Digit span forward/ reverse* Maximum digit span recorded [54], *F* Female, *GDA* Graded Difficulty Arithmetic [55], *GNT* Graded Naming Test [56], *IQ* Intelligence Quotient, *L* left, *lvPPA* Patient group with logopenic variant primary progressive aphasia, *M* Male, *MMSE* Mini Mental State Examination [57], *N* Normal, *NA*, Not available, *nfvPPA* Patient group with non-fluent variant primary progressive aphasia, *PALPA* Psycholinguistic Assessments of Language Processing in Aphasia [58], *PTA* Pure-tone average, *R* Right, *RMT* Recognition Memory Test [59], *Spatial span forward/ reverse* Maximum spatial span recorded [54], *svPPA* Patient group with semantic variant primary progressive aphasia, *Synonyms concrete/abstract* Single-word comprehension of single words [60], *tAD* Patient group with clinically typical Alzheimer’s disease, *Trail Making Test A*, Part A of the Trail Making Test [61], *VOSP* Visual Object Space Perception [62], *WASI* Wechsler Abbreviated Scale of Intelligence [63]

Mean (SD) values are shown. Raw scores are presented, with the maximum value possible given in parentheses, unless otherwise indicated; significant differences from healthy controls (*p* < 0.05) are shown in boldface type. Reduced numbers of participants completing particular tests were as indicated: ^a^*n* − 2; ^b^*n* − 1; ^c^*n* − 3; ^d^*n* − 4

^e^Note that tAD participants were given the short form of the RMT (maximum score of 25)

All participants gave informed consent for their involvement in the study. Ethical approval was granted by the University College London and National Hospital for Neurology and Neurosurgery Research Ethics Committees, in accordance with Declaration of Helsinki guidelines.

### Experimental stimuli and procedures

Two lists of spoken words were recorded, corresponding to two experimental conditions: 40 three-digit numbers (e.g., ‘nine hundred and sixty-five’) and 40 geographical place names (e.g., ‘Germany’). To reduce any crossover of perceptual learning effects based on intonational idiosyncrasies of particular speakers [32, 33], numbers were recorded by a young male speaker and place names by a young female speaker, both using a Standard English (southern England) accent. Place names comprised 20 cities and 20 countries, selected such that half of the items were located relatively ‘near’ (i.e., English cities, European countries) and half were more remote (i.e., relatively ‘far’ away: American cities, non-European countries). Inclusion of these ‘near’ and ‘far’ subcategories was intended to modulate the prior predictability of their sine-wave versions because geographical proximity has been shown to determine the relative familiarity of place names [23].

**Fig. 1:**
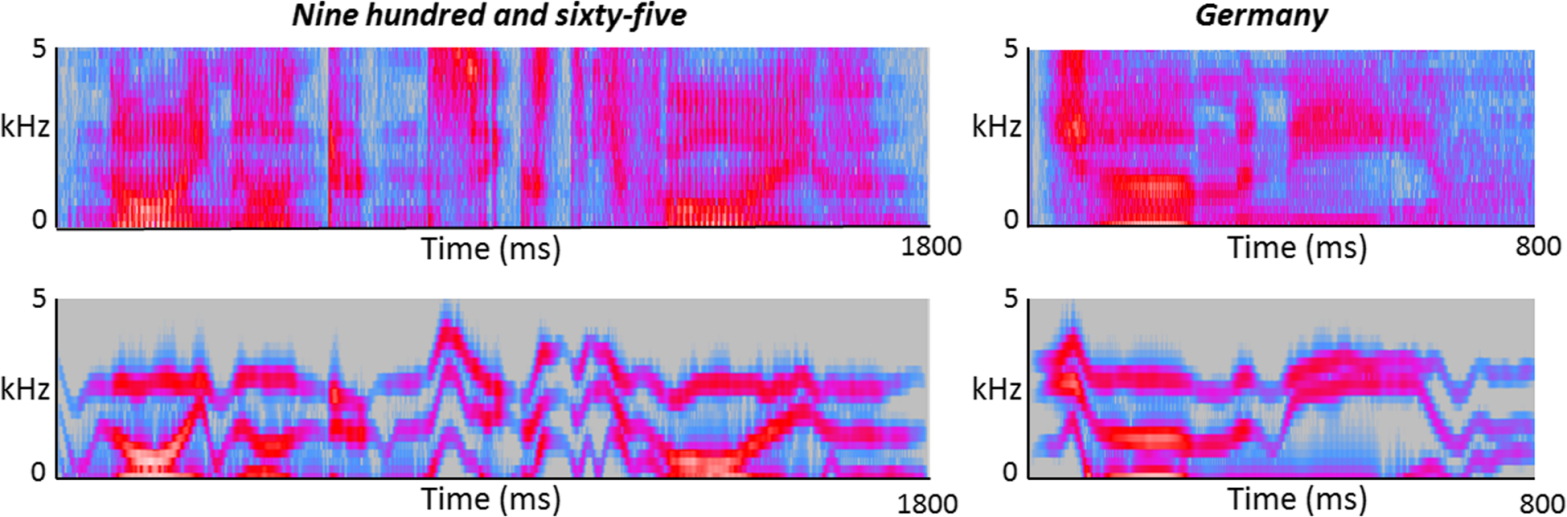
Broadband time-frequency spectrograms of sine-wave and natural speech. Examples of natural speech stimuli are shown in the top panels, and corresponding sine-wave replicas are shown in the bottom panels. The *y*-axes code frequency (kilohertz), and the *x*-axes code time (milliseconds). The centre frequencies of the three sine-wave contours track the centre frequencies of the formants in each of the natural stimuli. Depicted are examples of the two types of speech stimuli used in the experiment: three-digit numbers (‘nine hundred and sixty-five’; left panels) and geographical place names (‘Germany’; right panels). Sound recordings of these stimuli are available in Additional files 1, 2, 3, and 4

Sine-wave replicas of the natural speech recordings were generated using a procedure reported previously [15] (*see* Fig. 1). In creating the final stimulus lists, number stimuli were split into 2 blocks of 20 trials: the second block comprised 10 numbers that had been presented in the first block plus 10 numbers presented de novo. This design allowed us to assess the generalisability of any perceptual learning effects beyond the ‘trained’ stimulus set. Examples of stimuli wave files are provided in Additional files 1, 2, 3, and 4.

All stimuli were administered in a quiet room via headphones (ATH-M50x; Audio-Technica®, Leeds, UK) at a comfortable listening level (at least 70 dB) for each participant. The number condition was always presented before the place name condition (because we anticipated that any confounding, non-specific ‘training’ effects from prior sessional exposure would be more relevant to sine-wave number than place name identification [34]. The order of trials was fully randomised within each condition. Before commencing the test, participants were first familiarised with examples of natural spoken numbers and place names and advised that, during the test, similar words would be presented in distorted ‘whistled’ form and that these might initially be difficult to understand but might become easier over the course of the session. Participants were instructed that their task on each trial was to try to repeat and/or write down the distorted word as accurately as possible. During the test, no feedback about performance was given, and no time limits on responses were imposed.

Following the sine-wave conditions, two control conditions were administered to provide a measure of patients’ ability to process the verbal content of the number and geographical stimuli in natural speech. Ability to perceive speech under natural listening conditions was assessed by presenting a list of 10 undistorted three-digit numbers and a list of 16 undistorted place names. On each trial, the participant was required to repeat or transcribe the stimulus, and performance was scored similarly to the respective sine-wave conditions. In addition, geographical semantic knowledge of the 40 places presented during the sine-wave experiment was assessed using a two-alternative forced-choice procedure. For each place name (presented in undistorted form), participants were asked to indicate whether it was a city or a country; for cities, they were then asked to indicate whether it was English or American, and for countries, to indicate whether it was in Europe or elsewhere. An aggregate geographical knowledge score (total possible score of 80) was calculated for each participant. Numbers were scored per digit correct, meaning that each trial had a maximum score of 3, whereas place names were scored as either correct or incorrect.

### Analysis of clinical and behavioural data

Clinical and behavioural data were analysed using Stata version 14.0 software (StataCorp, College Station, TX, USA). Each patient group was compared with the healthy control participants using two-tailed, two-sample *t* tests for continuous variables and chi-square tests for categorical variables.

Analysis of variance (ANOVA) models were used to analyse participant group performance on all sine-wave and experimental control tests. Participants’ background geographical knowledge was analysed using total score on the geographical semantic control task as a dependent variable and participant group as an independent variable, covarying for general cognitive performance (Mini Mental State Examination [MMSE] score) to ensure that any group effects were not attributable simply to disease severity. The total score on this geographical knowledge index was then used as a covariate where indicated in subsequent analyses. In addition, one-tailed one-sample *t* tests were used within each diagnostic group separately to assess whether the performance difference between ‘near’ and ‘far’ trials was significantly greater than zero. Performance on the control tasks assessing natural speech perception was analysed for place names and numbers separately, incorporating syndromic diagnosis as an independent variable.

Participants’ overall perceptual decoding accuracy (identification) performance in each of the sine-wave speech conditions was analysed by incorporating syndromic diagnosis as an independent variable, with natural speech control task performance and (for place names) geographical semantic control task performance as covariates. The overall group effect of speech predictability (prior place name familiarity) was assessed using the difference between ‘near’ and ‘far’ location scores as the ANOVA dependent variable; post hoc *t* tests were used to compare this discrepancy index between participant groups. One-tailed one sample *t* tests were used within each diagnostic group separately to assess whether the difference between ‘near’ and ‘far’ trials was significantly greater than zero. In addition, to assess any condition-specific effects on sine-wave speech processing (and to capture potential variability of such effects between individuals), we calculated a ‘condition discrepancy index’ for each participant, defined as follows: ([score on sine-wave number condition/total score possible] minus [score on sine-wave geographical condition/total score possible]). This discrepancy index was analysed for any overall group effect as the ANOVA dependent variable with syndromic diagnosis as the independent variable; post hoc *t* tests were used to compare participant groups directly. One-sample *t* tests were used within each diagnostic group separately to assess whether the condition discrepancy index was significantly different from zero.

To assess change in participants’ performance with increasing exposure to sine-wave speech (auditory perceptual learning), we divided the number condition session into four blocks of ten trials and calculated a ‘perceptual learning index’ for each participant, defined as follows: ([block 4 score {trials 31–40}] minus [block 1 score {trials 1–10}]). Performance differences between initial and final stimulus presentation blocks have previously been shown to capture overall implicit learning of speech-like stimuli [16]. An analogous index was calculated for the place name condition. These learning index data were compared between participant groups using syndromic diagnosis as the ANOVA independent variable with natural speech task performance as a covariate. One-tailed one-sample *t* tests were used in each participant group separately to assess whether this perceptual learning index was significantly different from zero. Pearson’s correlations were used to assess any association of perceptual learning indices with MMSE score or Wechsler Abbreviated Scale of Intelligence (WASI) matrices score (proxies for global cognitive function), separately for sine-wave number and place name conditions in the combined patient cohort. For the sine-wave numbers condition, we also created a familiarity discrepancy index, defined as follows: (score on ‘trained’ [repeat] numbers minus score on ‘untrained’ [de novo] numbers). Participant groups were compared on this index using syndromic diagnosis as the ANOVA independent variable with natural speech task performance as a covariate. A threshold of *p* < 0.05 was accepted as the criterion for statistical significance in all tests.

### Brain image acquisition and analysis

Volumetric brain MRI scans were acquired for 33 patients in a 3-T MAGNETOM Prisma scanner (Siemens Healthcare, Erlangen, Germany) using a 64-channel head-and-neck receiver array coil and a T1-weighted sagittal 3D magnetization-prepared rapid gradient-echo sequence (echo time = 2.93 ms, inversion time = 850 ms, repetition time = 2000 ms), with matrix size 256 × 256 × 208 and voxel dimensions 1.1 × 1.1 × 1.1 mm and overall scan acquisition duration 306 seconds.

For the VBM analysis, patients’ brain images were pre-processed and normalised to Montreal Neurological Institute (MNI) space with isotropic voxel size 1.5 mm using SPM12 software (http://www.fil.ion.ucl.ac.uk/spm/software/spm12/) and the Diffeomorphic Anatomical Registration Through Exponentiated Lie Algebra (DARTEL) toolbox with default parameters in MATLAB R2014b (MathWorks, Natick, MA, USA), in accordance with a procedure we have described previously [14, 30]. Disease-associated atrophy profiles were generated for each patient group separately, again using a previously described protocol [30].

In parallel analyses over the combined patient cohort, separate linear regression models were implemented to assess associations between voxel-wise grey matter volume and (1) total score for sine-wave numbers, (2) total score for sine-wave place names, (3) sine-wave condition discrepancy indices (as defined above) and (4) sine-wave perceptual learning indices (as defined above). Each model incorporated symptom duration (as an index for disease stage), syndromic diagnosis, age and total intracranial volume as nuisance covariates. Statistical parametric maps were generated using an initial threshold *p* < 0.001 and assessed at peak statistical significance level *p* < 0.05, after family-wise error (FWE) correction for multiple voxel-wise comparisons within a pre-specified anatomical ROI. This region incorporated cortical areas in the dominant hemisphere that have been implicated in previous studies of degraded speech processing and auditory perceptual learning in the healthy brain (*see* Additional file 5), comprising the temporoparietal junction (including posterior superior temporal gyrus and sulcus, planum temporale, inferior parietal lobe), anterior temporal lobe and inferior frontal gyrus [28, 29, 35, 36], and an orofacial sensorimotor region encompassing the inferior two-thirds of the pre-central and post-central gyri [20].

## Results

Background demographic, neuropsychological and clinical data for all participant groups are presented in Table 1; group performance profiles on the experimental tasks are presented in Table 2.

**Table 2:** Performance of participant groups on experimental tasks

	Controls	nfvPPA	svPPA	lvPPA	tAD
Main sine-wave effect					
Numbers (total possible score of 120)	112.4 (6.1)	**53.1 (39.2)** ^a^	107.9 (9.2)	**57.6 (45.8)** ^a^	**98.4 (8.9)**
Place names (total possible score of 40)	35.4 (2.8)	**21.9 (11.4)**	**25.3 (4.4)**	**19.9 (11.7)**	**28.1 (5.6)**
Near − far place names	2.4 (3.2)^b^	**6.7 (4.3)** ^**b**^	**7.1 (2.4)** ^**b**^	**6.1 (2.8)** ^**b**^	**6.7 (3.5)** ^**b**^
Numbers > places	0.05 (0.1)^c^	− 0.10 (0.2)	**0.27 (0.1)** ^**c,d**^	− 0.02 (0.2)	0.12 (0.1)^c^
Perceptual learning effect					
Numbers	2.1 (3.2)^b^	3.1 (3.6)^b^	4.3 (3.5)^b^	3.7 (3.7)^b^	6.2 (6.3)^b^
Repeat − novel numbers	0.3 (2.1)	1.7 (3.3)	0.3 (2.6)	− 0.1 (1.6)	− 0.3 (1.6)
Place names	0.6 (1.2)^e^	2.2 (2.5)^e^	1.3 (3.4)	1.9 (2.3)^e^	− 0.1 (2.2)
Control tasks					
Natural speech numbers (total possible score of 30)	29.9 (0.2)	**21.1 (9.4)**	30.0 (0.0)	**22.1 (7.8)**	29.8 (0.4)
Natural speech place names (total possible score of 16)	16.0 (0.0)	**14.7 (0.6)**	15.9 (0.1)	**14.1 (0.9)**	16.0 (0.0)
Geographical knowledge (total possible score of 80)	79.9 (0.3)	75.8 (6.4)	**70.6 (12.0)** ^**d**^	73.1 (6.4)	75.6 (3.2)
Near − far place names	0.12 (0.3)	− 1.11 (3.4)	**4.0 (3.9)** ^**b**^	2.57 (2.2)^b^	0.2 (4.1)

*Abbreviations: Controls* Healthy control group, *lvPPA* Patient group with logopenic variant primary progressive aphasia, *Nat*. Natural, *nfvPPA* Patient group with non-fluent variant primary progressive aphasia, *svPPA* Patient group with semantic variant primary progressive aphasia, *SW* Sine wave, *tAD* Patient group with clinically typical Alzheimer’s disease

The table summarises participant group performance data for the key experimental tasks of interest assessing comprehension of sine-wave speech stimuli, natural speech and geographical semantic control tasks (*see text* for details). Perceptual learning indices were generated for each sine-wave condition by splitting the total number of trials in each condition into four trial blocks and subtracting block 1 score from block 4 score. Significant differences (*p* < 0.05) are coded as follows: bold, significant difference from healthy controls; ^a^significant difference from svPPA group; ^b^significant performance advantage for near > far places; ^c^significant within-group advantage for sine-wave numbers relative to place names; ^d^significant difference from all other participant groups; ^e^significant within-group improvement over time

### General participant group characteristics

Participant groups did not differ in age, handedness, gender, education or peripheral hearing (all *p* > 0.05). Patient groups differed on MMSE score (*p* = 0.034; less severe in svPPA and nfvPPA) and symptom duration (*p* = 0.026; shorter in nfvPPA and lvPPA).

### Experimental behavioural data

Performance on the natural speech control conditions differed significantly between participant groups for numbers [*F*(4,48) = 8.81, *p* < 0.001] and place names [*F*(4,47) = 5.52, *p* = 0.001]; post hoc comparisons between groups revealed that both the lvPPA and nfvPPA groups performed worse than healthy control participants and other patient groups (all *p* < 0.05) for both conditions. Performance on the geographical semantic control task was significantly affected by diagnosis [*F*(4,47) = 3.88, *p* = 0.008]. The svPPA group performed worse than all other participant groups (all *p* < 0.05); however, there were no other significant group performance differences on this task (all *p* > 0.05). In addition, the svPPA group (*t* = 3.28, *p* = 0.005) and the lvPPA group (*t* = 3.06, *p* = 0.011) showed a performance advantage for knowledge of ‘near’ over ‘far’ places; no other groups showed a significant performance discrepancy between trial types on this control task (all *p* > 0.05).

**Fig. 2:**
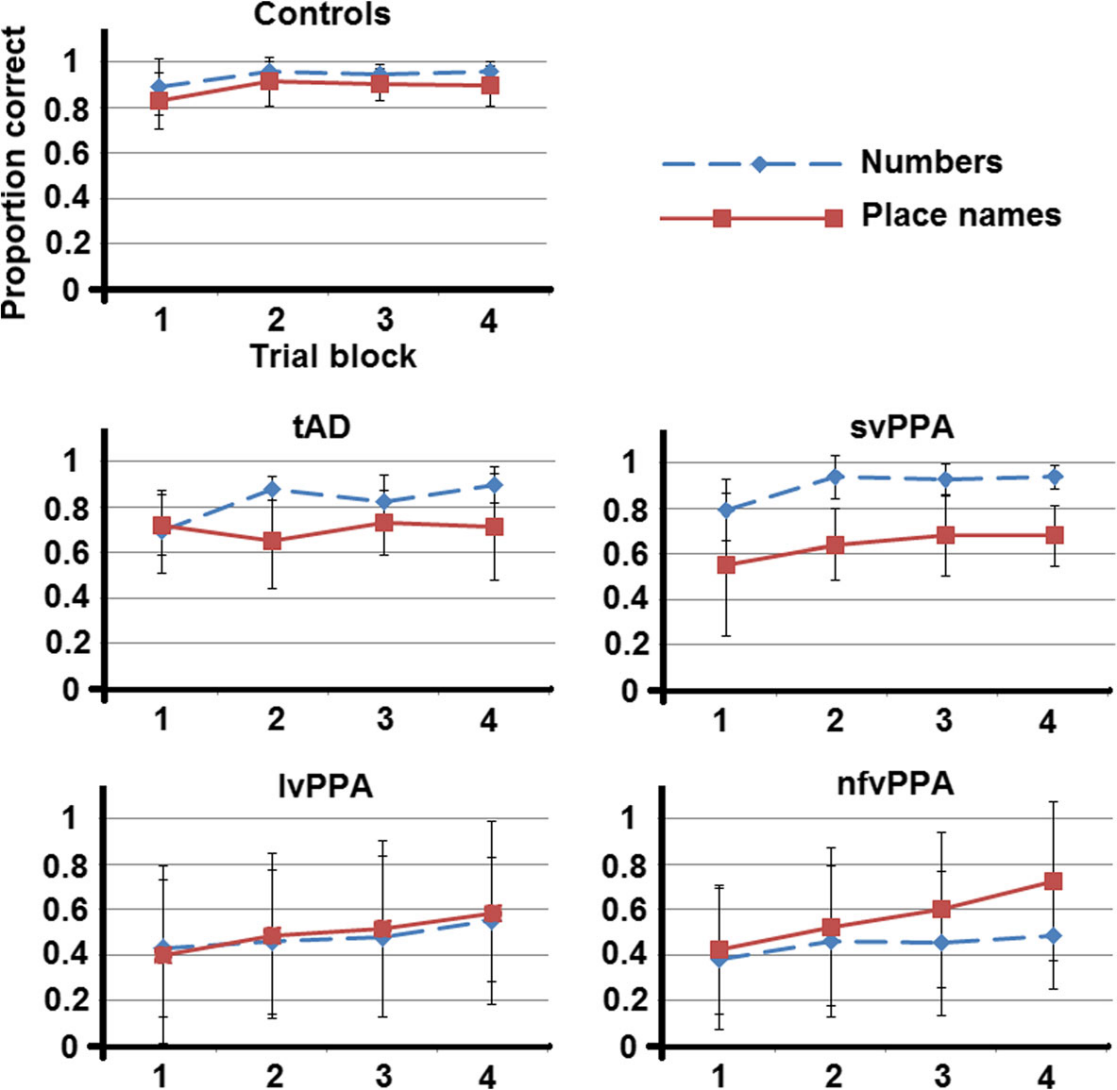
Participant group performance over testing sessions. Data plots of participant group performance over the course of the testing sessions for processing sine-wave replicas of spoken numbers and geographical place names. Values represent mean group scores with SDs for each corresponding trial block (*see text* for details) in each condition. *Controls* Healthy control group, *lvPPA* Patient group with logopenic variant primary progressive aphasia, *nfvPPA* Patient group with non-fluent variant primary progressive aphasia, *svPPA* Patient group with semantic variant primary progressive aphasia, *tAD* Patient group with clinically typical Alzheimer’s disease

Overall accuracy of perceptual decoding (identification) of sine-wave speech differed significantly between participant groups, for both the sine-wave number [*F*(4,47) = 3.91, *p* = 0.008] and place name [*F*(4,46) = 3.88, *p* = 0.009] conditions. For the sine-wave number condition, post hoc group comparisons revealed that the nfvPPA, lvPPA and tAD groups (but not the svPPA group) performed worse than healthy control participants (all *p* < 0.05) (Fig. 2). Comparing patient groups, both the lvPPA group (*t* = − 2.39, *p* = 0.021) and the nfvPPA group (*t* = − 2.53, *p* = 0.015) performed worse than the svPPA group. For the sine-wave place name condition, post hoc group comparisons revealed that all patient groups performed worse than healthy control participants (all *p* < 0.05), whereas there were no significant performance differences between patient groups. All participant groups showed a significant performance advantage for ‘near’ over ‘far’ place names (all *p* < 0.05), but the effect of place name type also differed significantly between participant groups [*F*(4,46) = 6.21, *p* = 0.004]; the advantage for ‘near’ over ‘far’ place names was significantly higher in each patient group relative to healthy control participants (all *p* < 0.05), but there were no differences between patient groups.

**Fig. 3:**
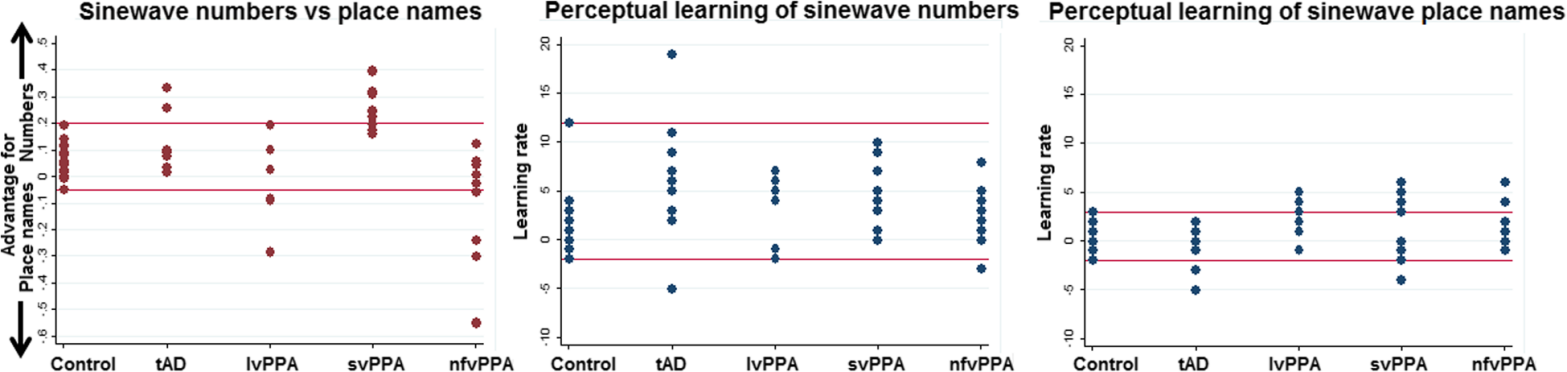
Individual performance. Scatterplots of individual performance on identification of sine-wave numbers relative to sine-wave place names (left), perceptual learning of sine-wave numbers (middle) and sine-wave place names (right). Sine-wave numbers vs place names (left panel) data were generated using the formula *y* = (sine-wave numbers score/total score possible) − (sine-wave places score/total score possible). Higher scores therefore indicate an advantage for identifying sine-wave numbers relative to sine-wave place names and vice versa. Perceptual learning data were generated by taking performance in block 1 away from block 4 for the sine-wave number (middle) and sine-wave place name (right) conditions. Red horizontal lines indicate the upper and lower bounds of the healthy control group range. *Controls* Healthy control group, *lvPPA* Patient group with logopenic variant primary progressive aphasia, *nfvPPA* Patient group with non-fluent variant primary progressive aphasia, *svPPA* Patient group with semantic variant primary progressive aphasia, *tAD* Patient group with typical Alzheimer’s disease

Comparing overall performance in the sine-wave number versus place name conditions in each participant group separately, the healthy control group (*t* = 3.70, *p* = 0.001), tAD group (*t* = 3.73, *p* = 0.002) and svPPA group (*t* = 9.93, *p* < 0.001) showed a significant performance advantage for identifying sine-wave numbers; there was no significant performance discrepancy between number and place name conditions in the lvPPA or nfvPPA groups (both *p* > 0.05). The performance condition discrepancy was significantly greater in the svPPA group than in each of the other groups (all *p* < 0.05). The svPPA group also showed the most individually consistent performance advantage for identifying sine-wave numbers: 80% of svPPA patients showed a performance discrepancy favouring numbers outside the healthy control range versus 20% of patients with tAD, whereas four patients with nfvPPA and three patients with lvPPA showed the reverse discrepancy, favouring sine-wave place name identification (*see* Fig. 3).

A significant within-group perceptual learning effect (indexed as block 4 score minus block 1 score) was evident for the sine-wave number condition in all participant groups (*p* < 0.05). This perceptual learning index was positively correlated with block 1 score across participants (*p* = 0.027). There was no difference between repeat versus new items over the combined participant cohort [*F*(4,47) = 2.29, *p* = 0.073] or the combined patient cohort [*F*(3,31) = 2.80, *p* = 0.056]. For the sine-wave place name condition, the healthy control, lvPPA and nfvPPA groups showed a significant perceptual learning effect (all *p* < 0.05), whereas the svPPA and tAD groups showed no such effect. However, there was no significant overall group effect on perceptual learning for the sine-wave number condition [*F*(4,47) = 1.79, *p* = 0.147] or place name condition [*F*(4,46) = 1.80, *p* = 0.144]. Inspection of individual performance data (Fig. 3) showed that for sine-wave numbers, only two patients (one tAD, one nfvPPA) had a perceptual learning rate below the lower bound seen in the healthy control group, whereas for sine-wave place names, two patients with tAD and one patient with svPPA scored lower than the control range. Perceptual learning index was not significantly correlated with MMSE score or WASI matrices score for either the number condition (MMSE, *r* = 0.001, *p* = 0.995; WASI matrices, *r* = − 0.059, *p* = 0.731) or place name condition (MMSE, *r* = 0.029, *p* = 0.863; WASI matrices, *r* = 0.109, *p* = 0.528) across the patient cohort.

### Neuroanatomical data

**Fig. 4:**
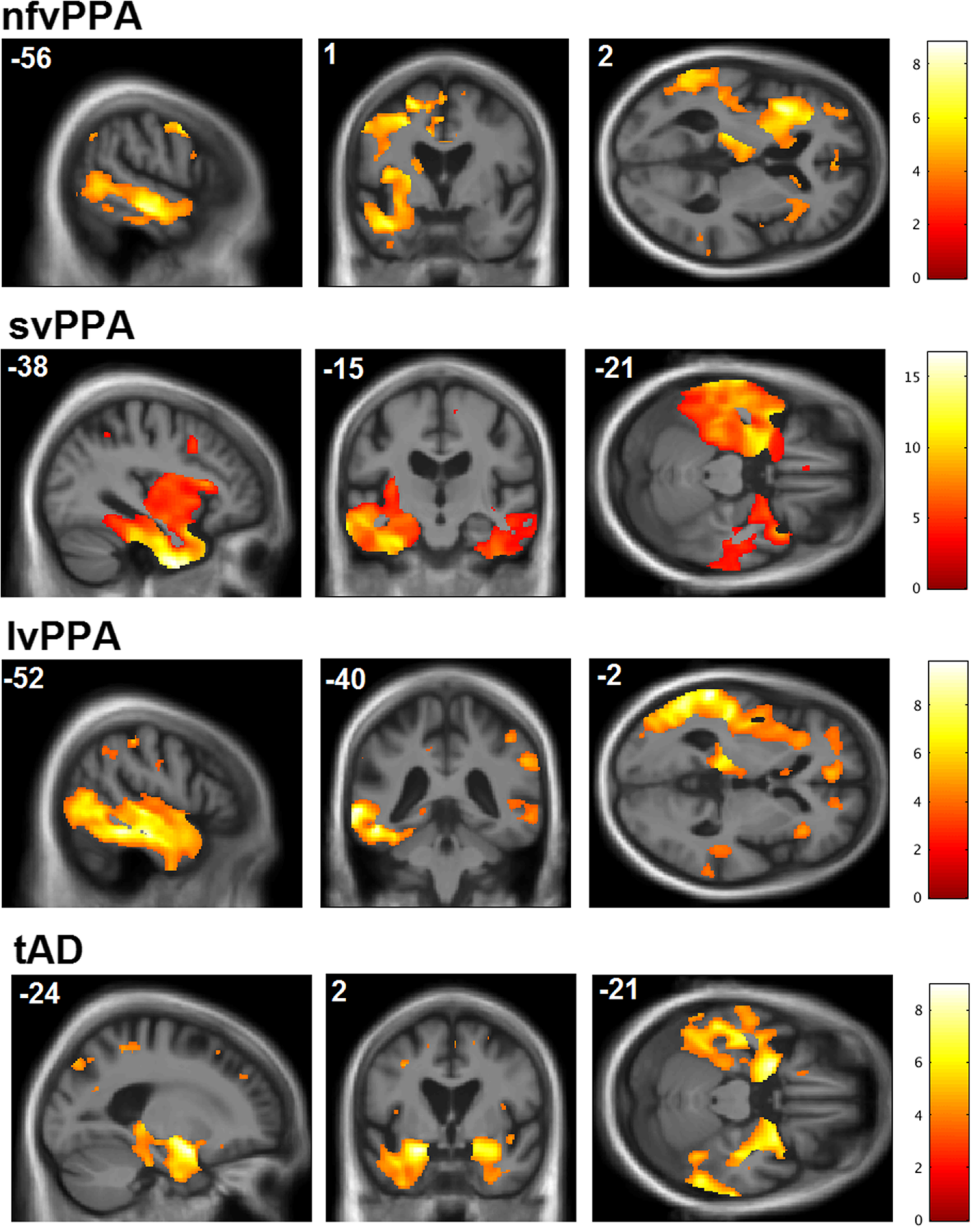
Statistical parametric maps showing disease-related structural neuroanatomical differences between each patient group and controls. Maps are thresholded at *p* < 0.001 uncorrected over the whole brain and displayed on representative sections of a group (combined patient cohort) mean T1-weighted brain magnetic resonance image; the plane of each section is indicated using Montreal Neurological Institute (MNI) coordinates, and the left cerebral hemisphere is displayed on the left in coronal sections and on top in axial sections (colour bars code voxel-wise *t* scores for the relevant atrophy map). *nfvPPA* Non-fluent variant primary progressive aphasia, *svPPA* Semantic variant primary progressive aphasia, *lvPPA* Logopenic variant primary progressive aphasia, *tAD* Typical Alzheimer’s disease

**Fig. 5:**
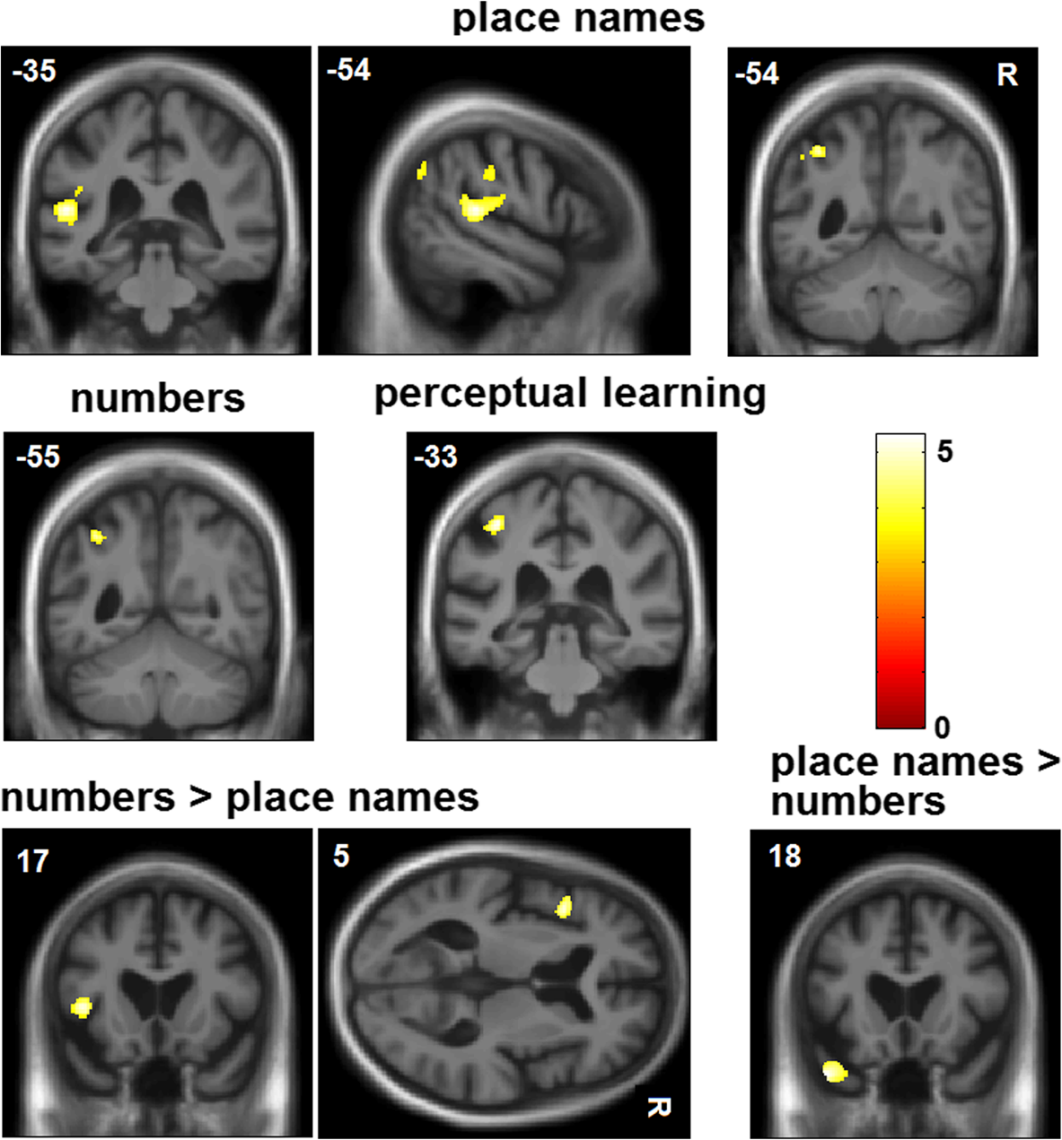
Voxel-based morphometry results. Statistical parametric maps of regional grey matter volume positively associated with performance on sine-wave speech processing tasks for the combined patient cohort. The top panels show grey matter correlates of sine-wave number and place name identification accuracy; the middle panels, correlates of significant performance discrepancy between the sine-wave conditions (performance advantage for sine-wave numbers or place names); the bottom panels, correlates of the perceptual learning effect over the sine-wave number session (se*e text* for details). Maps are rendered on sections of the group mean T1-weighted brain magnetic resonance image, thresholded at *p* < 0.001 uncorrected for multiple voxel-wise comparisons over the whole brain for display purposes (areas shown were significant at *p* < 0.05_FWE_ for multiple comparisons within a pre-specified neuroanatomical ROI; *see* Table 3 and Additional file 5). The left hemisphere is presented on the left for coronal sections and on top in the axial section; Montreal Neurological Institute (MNI) coordinates for the plane of each section are indicated. The colour bar codes voxel-wise *t* scores for each map

Patient groups showed the anticipated syndromic profiles of disease-related grey matter atrophy; statistical parametric maps are presented in Fig. 4. Statistical parametric maps of grey matter regions associated with performance on the sine-wave speech-processing tasks are shown in Fig. 5; local grey matter maxima associated with performance on each variable of interest are summarised in Table 3. All contrasts describing associations with behavioural data are reported after FWE correction for multiple voxel-wise comparisons within the pre-specified neuroanatomical ROI.

**Table 3:** Structural neuroanatomical associations of sine-wave speech comprehension in the patient cohort

Contrast	Region	Cluster (voxels)	Peak (mm)			*t* Score	*p* Value
			*x*	*y*	*z*		
Sine-wave numbers	Angular gyrus	12	− 38	− 54	46	4.63	0.045
Sine-wave place names	Planum temporale	377	− 52	− 34	12	5.19	0.013
	Angular gyrus	25	− 39	− 54	48	4.74	0.035
Place names > numbers	Temporal pole	383	− 46	18	− 34	5.03	0.018
Numbers > place names	Inferior frontal gyrus	84	− 45	16	4	4.70	0.037
Perceptual learning: sine-wave numbers	Post-central gyrus	175	− 38	− 33	54	5.26	0.011
	Post-central gyrus	627	− 58	− 20	38	4.96	0.021

The table summarises statistically significant (positive) associations between regional grey matter volume and the relevant performance measure for the processing of sine-wave speech stimuli (*see text* for details), based on a voxel-based morphometric analysis of brain magnetic resonance images for the combined patient cohort. All local maxima presented are significant at *p* < 0.05_FWE_ within a pre-specified left hemispheric ROI (*see* Additional file 5). Coordinates of local maxima are in Montreal Neurological Institute (MNI) standard space

Across the combined patient cohort, identification of sine-wave numbers was significantly positively associated with grey matter volume in the left angular gyrus (*p* = 0.045_FWE_), whereas identification of sine-wave place names was positively associated with grey matter volume in the left planum temporale (*p* = 0.013_FWE_) and left angular gyrus (*p* = 0.035_FWE_). A performance advantage for sine-wave numbers over place names was significantly positively associated with grey matter volume in the left inferior frontal gyrus (*p* = 0.037_FWE_), whereas the inverse condition discrepancy effect (performance advantage for sine-wave place names over numbers) was significantly positively associated with grey matter volume in the left temporal pole (*p* = 0.018_FWE_). The perceptual learning index for sine-wave numbers was significantly positively associated with grey matter volume in the left inferolateral post-central gyrus (two peaks at *p* = 0.011_FWE,_ *p* = 0.021_FWE_). There were no significant grey matter associations of perceptual learning for sine-wave place names over the combined patient cohort.

## Discussion

In the present study, we have demonstrated deficits of degraded speech perception in major syndromes of PPA and tAD relative to healthy control participants. Syndromic groups were stratified by their overall accuracy in decoding sine-wave speech, with patients with nfvPPA and lvPPA exhibiting the most severe and consistent impairments. The findings in tAD extend previous evidence in this disease [15]. Syndromic profiles were modulated by the prior predictability of verbal content: The svPPA and tAD groups showed a significant advantage for perceiving sine-wave speech with highly predictable content (numbers) compared with less predictable content (place names), whereas within the geographical condition, all groups showed a performance advantage for more familiar (‘near’) over less familiar (‘far’) place names, and this advantage was exaggerated in patient groups compared with the healthy control participants. All syndromic groups exhibited some capacity for auditory perceptual learning following sustained exposure to sine-wave speech: this occurred spontaneously, and there was evidence that the effect generalised to ‘new’ as well as ‘trained’ speech tokens. However, patients with svPPA and tAD showed a perceptual learning effect only for relatively predictable verbal content. These effects were evident after adjusting for performance on natural speech perception and geographical knowledge tasks and therefore not attributable to more generic deficits of phonological or semantic processing in the patient groups. Taken together, the present results imply a degree of residual cerebral plasticity in these syndromes, manifesting as resilient auditory perceptual learning of degraded speech.

Our finding that decoding of degraded speech is impaired in the nfvPPA, lvPPA and tAD groups corroborates recent evidence for core auditory processing deficits in these syndromes [5–8, 10, 14, 27, 30]. The less uniform decoding deficit identified in the svPPA group in the present study was also anticipated on the basis of previous work; in the healthy brain, decoding of degraded speech engages ‘top-down’ (including semantic) mechanisms that disambiguate the speech stream based on prior predictability [10, 13, 37]. Efficient access to stored semantic ‘priors’ (including ready access to lower-frequency priors) when interpreting degraded speech is likely to become increasingly limiting as verbal content becomes less predictable [26]; less predictable verbal content would place increased demands on semantic processing resources. This would account both for the striking performance advantage for (highly predictable) sine-wave numbers over (less predictable) place names shown in this study by patients with svPPA and for the ‘echo’ of this condition discrepancy effect in the performance advantage for more familiar over less familiar place names shown by all participant groups. This was not simply the consequence of a dwindling semantic lexicon; it was observed after taking geographical semantic competence into account, in keeping with a more specific limitation on the recruitment of semantic mechanisms during predictive processing of speech signals. Furthermore, the tAD group, but not the lvPPA group, showed a significant condition discrepancy effect, suggesting that these Alzheimer variant syndromes may be characterised by separable pathophysiological mechanisms [2].

Participant groups did not differ in perceptual learning of sine-wave numbers. Indeed, the magnitude of the perceptual learning effect across patient groups (including those with marked overall deficits of degraded speech perception) was comparable to that of healthy older control participants (Table 2, Figs. 2 and 3). Moreover, most individual patients in each syndromic group showed perceptual learning effects within the healthy control range. There was no association between general cognitive performance (indexed by MMSE and WASI matrices scores) and perceptual learning index for numbers or place names. Together, these findings argue for dissociable physiological mechanisms mediating the accuracy of degraded speech decoding and adaptation based on sustained, unsupervised exposure to degraded speech, and they suggest that perceptual learning of degraded speech may be relatively resilient to the effects of background cognitive decline. The syndromic profiles in the present study further suggest that perceptual learning is modulated by prior verbal predictability (the svPPA and tAD groups showed the effect for strongly predictable but not less predictable verbal stimuli), in line with current models of degraded speech learning based on minimising prediction errors [13]. Although data in PPA are limited, our findings are consistent with previous work in nfvPPA, indicating that separable mechanisms underpin decoding of sensory detail in degraded speech stimuli, ‘top-down’ predictions about such stimuli and implicit learning based on auditory experience [10, 16]. There are precedents for a dissociation of sensory accuracy and sensory learning or plasticity in other disorders (e.g., developmental dyslexia and amblyopia [38]), with potential substrates at cognitive, neurophysiological and neuroanatomical levels [39]. Speech may be a particularly potent stimulus to expose the component mechanisms of a processing hierarchy. Whereas perceptual decision making on speech and other complex auditory stimuli typically rests on integration of multiple spectrotemporal features, perceptual learning may depend on the extraction of more specific lower-level properties from ‘bottom-up’ sensory data, honed by ‘top-down’ predictions based on prior auditory experience and used in turn to update those predictions [13, 39]. This reciprocal interaction between sensory traffic and predictions could be instantiated on different neuroanatomical scales, ranging from local cortical circuits to large-scale distributed brain networks that could be differentially disrupted by neurodegenerative proteinopathies [10, 17, 40].

The neuroanatomical correlates of degraded speech decoding accuracy and perceptual learning identified in our patient cohort support the behavioural evidence that these processes are at least partly dissociable. Identification of both sine-wave numbers and place names was associated with grey matter volume in left angular gyrus, a region affected by the neurodegenerative pathologies studied here [41, 42] and previously implicated in processing speech under challenging listening conditions in functional neuroimaging and virtual lesion studies in the healthy brain [17, 43–46]. The present evidence in a patient cohort with variably impaired perception of degraded speech corroborates this previous work and further suggests that integrity of angular gyrus plays a critical role in determining whether degraded speech is disambiguated successfully. However, this region acts as the hub of a distributed processing network. Its functional connectivity and interactions with other modes of the network may be modulated by a number of factors, including output task, semantic context and perceived intelligibility [47]. In line with this, we identified additional neuroanatomical correlates that may mediate the effect of altered verbal predictability of degraded speech (summarised in Table 3). Grey matter in the left planum temporale was correlated with identification of sine-wave place names but not numbers at the prescribed threshold. This region is engaged in parsing the auditory scene under conditions of high computational load [13, 28, 36].

Candidate loci for ‘top-down’ control of perceptual analysis under different conditions of verbal predictability were identified in the left inferior frontal gyrus (for more highly predictable, sine-wave numbers) and left temporal pole (for less predictable sine-wave place names). Our findings do not resolve the mechanisms by which these regions communicate during sine-wave speech decoding, which are likely to differ between dementia syndromes. Disambiguation of degraded speech based on relatively constrained predictive algorithms (such as word verification or number identification) is likely to engage top-down control mechanisms in the inferior frontal cortex, a region that is heavily involved in nfvPPA [10], whereas decoding of ‘novel’ linguistic environments with less predictable verbal content (such as degraded place names) may demand active computation of the ‘best fit’ between incoming speech signal statistics and stored verbal concepts, accessed via the anterior temporal lobe semantic network that is blighted in svPPA [14, 26, 30, 48]. Considered together, our findings suggest that the overall accuracy of degraded speech decoding in these syndromes is likely to depend on a distributed peri-Sylvian network closely overlapping classical language cortices. Both nfvPPA and lvPPA (and less consistently svPPA) have been shown to be associated with atrophy or dysfunction of the dorsal language network, with involvement of anterior and posterior regions extending beyond the zone of maximal atrophy in particular syndromes [2, 5]. Impaired accuracy of sine-wave speech identification might plausibly result from a ‘double-hit’ to inferior frontal and temporoparietal regions previously implicated in decoding speech and other complex auditory signals [4, 5, 30].

A neuroanatomical substrate for perceptual learning of degraded speech (sine-wave numbers) was identified in the inferolateral post-central gyrus. This sector of sensorimotor cortex does not form part of the canonical language network and was not associated with sine-wave speech identification accuracy in this study, consistent with dissociable neural mechanisms for perceptual decision making and perceptual learning of degraded speech. However, this sensorimotor region hosts cortical representations of lips, mouth and tongue that are engaged during speech perception, particularly under difficult listening conditions in which subvocal rehearsal may help to resolve ambiguous speech sounds [13, 20] or in the context of disease processes primarily affecting the auditory cortex [21]. Sensorimotor cortices are relatively spared in PPA syndromes and tAD [1, 49]; we propose that a critical determinant of perceptual learning capacity (cerebral plasticity) is the degree of atrophy (or relative preservation) of these areas. Our findings suggest that some degree of residual neural plasticity is maintained beyond vulnerable language and auditory networks across canonical dementia syndromes. A stronger claim would be that some form of compensatory functional enhancement drives perceptual learning in the face of neurodegenerative pathology. We cannot evaluate this claim on the basis of the present evidence, though we note that no patient group in our study showed *increased* perceptual learning capacity relative to healthy older control participants. To understand the nature of the observed effects fully will require functional neuroimaging approaches that can address network connectivity and activity changes directly. Functional neuroimaging paradigms based on degraded speech stimuli have been developed in the healthy brain [13, 50], but they have yet to be applied to patients with dementia. The present findings corroborate our previous psychoacoustic work in tAD [15] and the recent demonstration that patients with nfvPPA benefit from retraining strategies for speech production and fluency, with lasting and generalisable improvement of communication function [51]. Our findings raise the further intriguing possibility that the efficacy of such communication interventions may be enhanced by engaging perceptual learning capacity (i.e., residual cerebral plasticity). If we are to exploit this potential, quantitative behavioural and neuroanatomical markers of perceptual learning will be required. Sine-wave speech (and related degraded speech manipulations) may offer a convenient and well-established route to development of relevant plasticity biomarkers.

This study suggests a number of directions for future work. Sine-wave speech served in the present study as a model paradigm for understanding speech under challenging listening conditions. We found that capacity for perceptual learning of this radically degraded speech-like signal is retained across diverse dementia syndromes, despite variably impaired understanding of the signal. Measures of individual responses (Fig. 3) suggest that these stimuli may represent novel markers for assessing and tracking communication function in particular patients and could have therapeutic potential. Dynamic markers of this kind might stratify dementia syndromes but also transcend conventional syndromic boundaries, constituting ‘stress tests’ of speech processing and auditory scene analysis in earlier-stage PPA and tAD and also presenting a target for intervention. This need not await the advent of disease-modifying therapies; combining currently available symptomatic pharmacotherapies (such as cholinesterase inhibitors) with speech retraining might be one rational approach [15, 16]. However, more information is required about the diagnostic sensitivity, specificity and relevance of perceptual measures on degraded speech over the course of disease, based on replication of these findings in larger patient cohorts, correlation with indices of daily life communication functions, and extension to other speech manipulations. In addition, detailed understanding of the pathophysiological mechanisms of degraded speech processing and perceptual learning in neurodegenerative syndromes will require functional neuroimaging and connectivity-based techniques that can capture activity profiles and time-varying interactions between brain regions.

## Conclusions

This work has broad neurobiological and clinical implications. Neurobiologically, the findings suggest that neurodegenerative proteinopathies dissect dissociable mechanisms for auditory pattern decoding and adaptation and expose the critical brain substrates for these processes. Clinically, this work forecasts a fresh emphasis on dynamic physiological capacity and functional plasticity in dementia that should motivate novel biomarker development and neurorehabilitation strategies.

## Additional files

Additional file 1: Clear place name. Natural speech version of SJR saying “Germany”. (WAV 99 kb)

Additional file 2: Sine-wave place name. Sine wave speech version of SJR saying “Germany”. (WAV 77 kb)

Additional file 3: Clear number. Natural speech version of CJDH saying “Nine hundred and sixty five”. (WAV 201 kb)

Additional file 4: Sine-wave number. Sine-wave speech version of CJDH saying “Nine hundred and sixty five”. (WAV 163 kb)

Additional file 5: ROIs. Representative brain MRI sections showing the neuroanatomical region (delineated in red) used to correct for multiple voxel-wise comparisons, based on prior anatomical hypotheses (*see text*). This region comprised the inferior frontal gyrus (triangularis + opercularis), anterior temporal lobe, temporal pole, posterior superior temporal gyrus, planum temporale, angular gyrus, supramarginal gyrus and inferior portions of the pre-central and post-central gyri. (PNG 138 kb)

## Abbreviations

ANOVA: Analysis of variance
DARTEL: Diffeomorphic Anatomical Registration Through Exponentiated Lie Algebra
FWE: Family-wise error
lvPPA: Logopenic variant primary progressive aphasia
MMSE: Mini Mental State Examination
MNI: Montreal Neurological Institute
MRI: Magnetic resonance imaging
nfvPPA: Non-fluent variant primary progressive aphasia
PPA: Primary progressive aphasia
svPPA: Semantic variant primary progressive aphasia
tAD: Typical Alzheimer’s disease
VBM: Voxel-based morphometry
