# Supplementary figures and images for: Retained capacity for perceptual learning of degraded speech in primary progressive aphasia and Alzheimer’s disease
Source: Alzheimers Res Ther. 2018 Jul 25;10:70. doi: 10.1186/s13195-018-0399-2 (PMC6060531; doi:10.1186/s13195-018-0399-2)

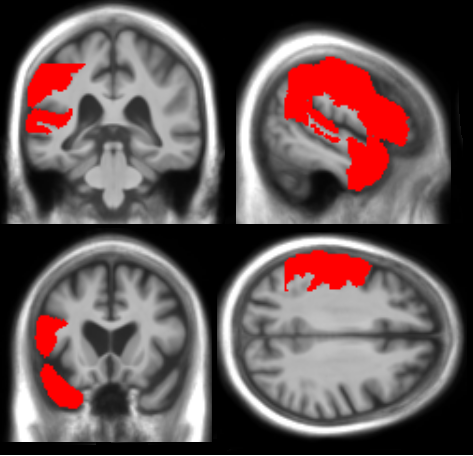

Supplement: Supplementary file 5 — ROIs. Representative brain MRI sections showing the neuroanatomical region (delineated in red) used to correct for multiple voxel-wise comparisons, based on prior anatomical hypotheses (see text). This region comprised the inferior frontal gyrus (triangularis + opercularis), anterior temporal lobe, temporal pole, posterior superior temporal gyrus, planum temporale, angular gyrus, supramarginal gyrus and inferior portions of the pre-central and post-central gyri. (PNG 138 kb) [file 13195_2018_399_MOESM5_ESM.png]
